# Zero-Dimensional Lead-Free Perovskite Single Crystals for High-Performance Photodetection

**DOI:** 10.3390/nano16211402

**Published:** 2026-11-01

**Authors:** Jiayu Di, Shuixiu Lyu, Liuyi Li, Dawei Wang, Bin Lu

**Affiliations:** 1 School of Computer Science and Artificial Intelligence, Shanxi Normal University, Taiyuan 030031, China; 2 School of Physics and Electronic Engineering, Shanxi Normal University, Taiyuan 030031, China

**Keywords:** zero-dimensional, lead-free, single crystal, photodetector

## Abstract

The rapid development of optoelectronic technologies has placed ever-increasing demands on high-performance photodetectors, as the intrinsic properties of the active materials fundamentally determine the detection limit and operational stability. Although organic–inorganic hybrid perovskites exhibit superior optoelectronic properties, lead-based perovskites suffer from the drawbacks of heavy-metal toxicity and poor structural stability, which severely limit their practical applications. Low-dimensional lead-free perovskite single crystals have therefore emerged as a promising material system, owing to their extremely low defect density, grain-boundary-free nature, and strong intrinsic stability. Here, high-quality 0D lead-free MA_3_Sb_2_I_9_ single crystals were grown via a seed-induced two-step crystallization method, and photodetectors based on these single crystals were fabricated. The resulting photodetector exhibits a high responsivity of 22.77 A/W, an external quantum efficiency (EQE) of 6971.92%, and a specific detectivity of 1.49 × 10^12^ Jones. Notably, stable photoresponse was maintained over ~450 on/off switching cycles, evidencing excellent operational repeatability and long-term stability. These results highlight the great potential of MA_3_Sb_2_I_9_ single crystals as a stable, lead-free candidates for high-performance photodetection.

## 1. Introduction

The rapid advances in optoelectronic integration, intelligent sensing, and high-speed optical communication have imposed ever-stricter demands on high-performance photodetectors [1,2,3]. Among these, the intrinsic properties of the optoelectronic functional materials are key to determining the detection limit and operational stability of photodetectors. Organic–inorganic hybrid perovskites, owing to their superior optoelectronic properties and extremely low fabrication cost, have been widely studied and reported in optoelectronic fields such as photodetection and solar cells [4,5]. Research on perovskite photodetectors is largely focused on polycrystalline thin-film systems, where existing studies mainly employ additive engineering, surface passivation, and post-annealing treatment to suppress film defects and optimize optoelectronic performance [6,7,8].

Meanwhile, conventional lead-based perovskites suffer from inherent drawbacks such as heavy-metal toxicity and poor structural stability [9], which hinder their use in environmentally friendly devices. To address these challenges, low-dimensional lead-free perovskite single crystals, owing to their unique advantages of extremely low defect density, grain-boundary-free nature, and strong intrinsic stability [10,11,12], have gradually emerged as a preferred material system in the field of photodetection. Typical representatives are Sn-based lead-free perovskites [13,14]. Nevertheless, the oxidative instability of Sn^2+^ remains a key limitation [15,16], motivating the exploration of more robust Sb-based alternatives such as MA_3_Sb_2_I_9_. Compared with Sn-based systems, Sb-based perovskites offer better environmental stability. However, photodetection performance of 0D MA_3_Sb_2_I_9_ single crystals remains underexplored.

(CH_3_NH_3_)_3_Sb_2_I_9_ (MA^+^ = CH_3_NH_3_^+^, MA_3_Sb_2_I_9_) is a typical lead-free antimony-based perovskite single-crystal material [17], which not only fundamentally circumvents the biotoxicity and environmental pollution issues of lead-based perovskites from the source, but also effectively resolves the drawbacks of Sn-based lead-free systems, namely their susceptibility to oxidation and poor stability [18,19]. Compared with polycrystalline thin films [20], MA_3_Sb_2_I_9_ single crystals feature a complete lattice structure and an extremely low defect density, which can suppress carrier recombination losses and thereby enhance the response speed and detection sensitivity of photodetectors. Furthermore, their superior optical stability and environmental tolerance render them highly promising for high-precision photodetection applications. At present, the controllable preparation of high-quality MA_3_Sb_2_I_9_ single crystals and the construction of high-performance photodetector devices still require further exploration.

In this work, the lead-free zero-dimensional (0D)-structured MA_3_Sb_2_I_9_ perovskite was selected as the target material, and high-quality single crystals were grown by a seed-induced two-step crystallization method. The photodetector based on the MA_3_Sb_2_I_9_ single crystal exhibited a high responsivity of 22.77 A/W, an external quantum efficiency (EQE) of 6971.92%, and a detectivity of 1.49 × 10^12^ Jones. Moreover, no attenuation of the photoresponse was observed during repeated on/off switching tests (~450 cycles), demonstrating excellent operational repeatability and long-term stability.

## 2. Materials and Methods

### 2.1. Single-Crystal Preparation

#### 2.1.1. Materials

2.72 g MAI (Methylammonium iodide, Shanghai Macklin Biochemical Co., Ltd., Shanghai, China, ≥99.5%) and 5.68 g SbI_3_ (Antimony (III) iodide, ultra-dry, Shanghai Macklin Biochemical Co., Ltd., Shanghai, China, 99.9999%) were dissolved in 20 mL GBL (γ-Butyrolactone, Sigma-Aldrich, Shanghai, China, ≥99%) at a molar ratio of 3:2 to generate a ~0.28 M MA_3_Sb_2_I_9_ precursor solution. The precursors were stirred at a constant rate of 300 rpm in the atmospheric environment for 24 h at 70 °C. After filtration with a 0.45 μm organic filter head, the precursor solution was clarified and transferred to a pre-cleaned crystallizing dish for subsequent use.

#### 2.1.2. Methods

**Figure 1:**
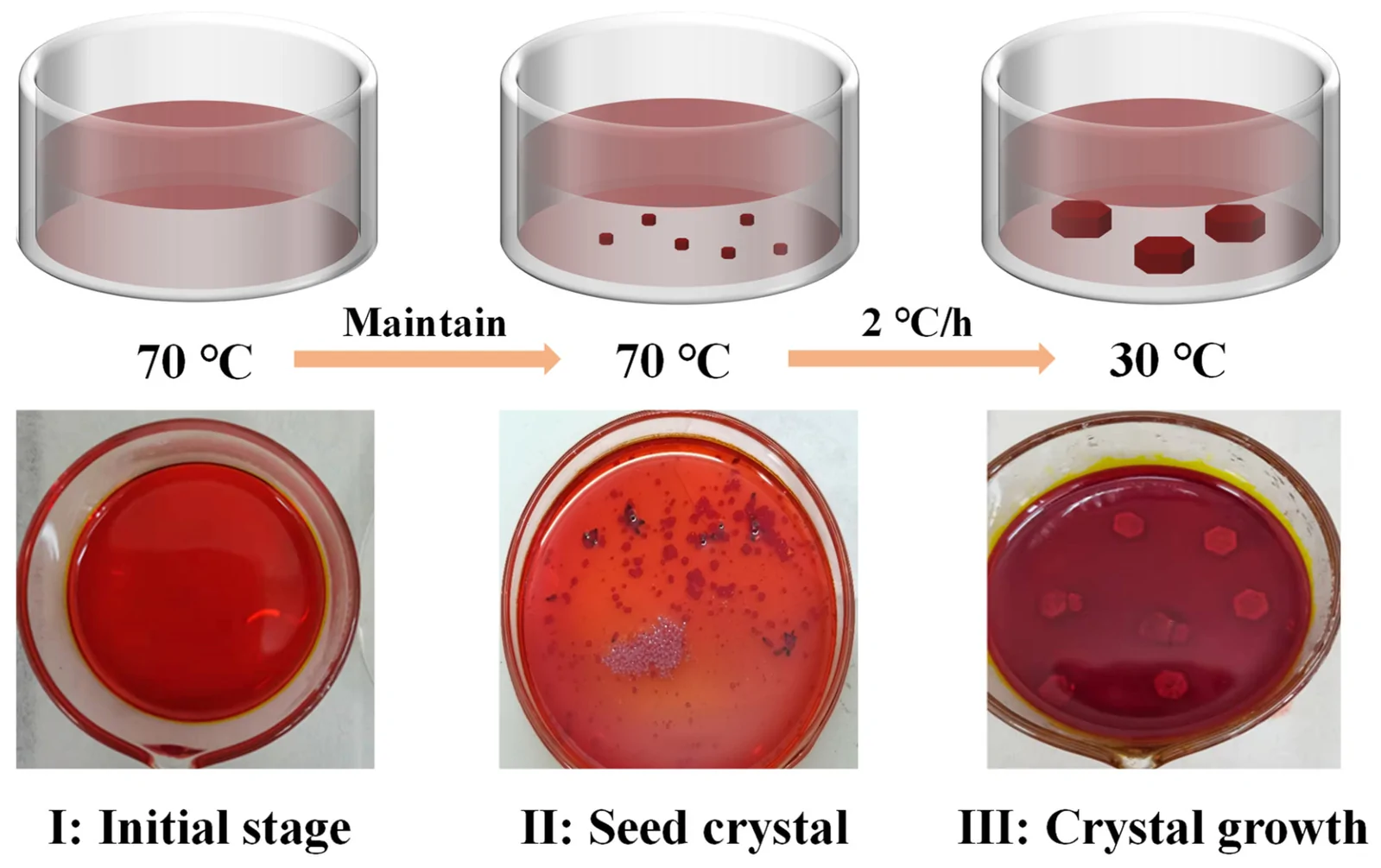
Schematic diagrams and corresponding photograph of the growth process of MA_3_Sb_2_I_9_ perovskite single crystals.

Given the low solubility of inorganic SbI_3_ in GBL, a seed-induced two-step crystallization method was employed to synthesize high-quality 0D MA_3_Sb_2_I_9_ perovskite single crystals. Figure 1 schematically illustrates the entire preparation process, along with the corresponding optical photographs at each growth stage. The first step involved the preparation and screening of seed crystals. The saturated precursor solution was maintained at a constant temperature of 70 °C for 12 h to ensure complete dissolution. And the crystallization dish was sealed with parafilm pierced by a small pinhole to isolate dust and airborne impurities, prevent crystal contamination, and control the solvent evaporation rate, thereby building a stable environment for crystal nucleation. This treatment also slowed down the solvent evaporation rate to build a stable environment for crystal nucleation. Uniform nucleation was achieved through controlled spontaneous solvent evaporation, yielding initial seed crystals. Seed crystals with regular morphology, few defects and uniform size were selected as high-quality seeds for subsequent single-crystal growth. The second step was the growth of large-size single crystals. The screened high-quality seed crystals were transferred into a freshly prepared saturated precursor solution. A slow cooling crystallization strategy was adopted, in which the temperature was gradually decreased to 30 °C at a constant cooling rate of 2 °C/h, thereby driving continuous and uniform growth of the seed crystals. Ultimately, large-size 0D MA_3_Sb_2_I_9_ perovskite single crystals with excellent crystallinity and low defect density were successfully obtained.

## 3. Results

**Figure 2:**
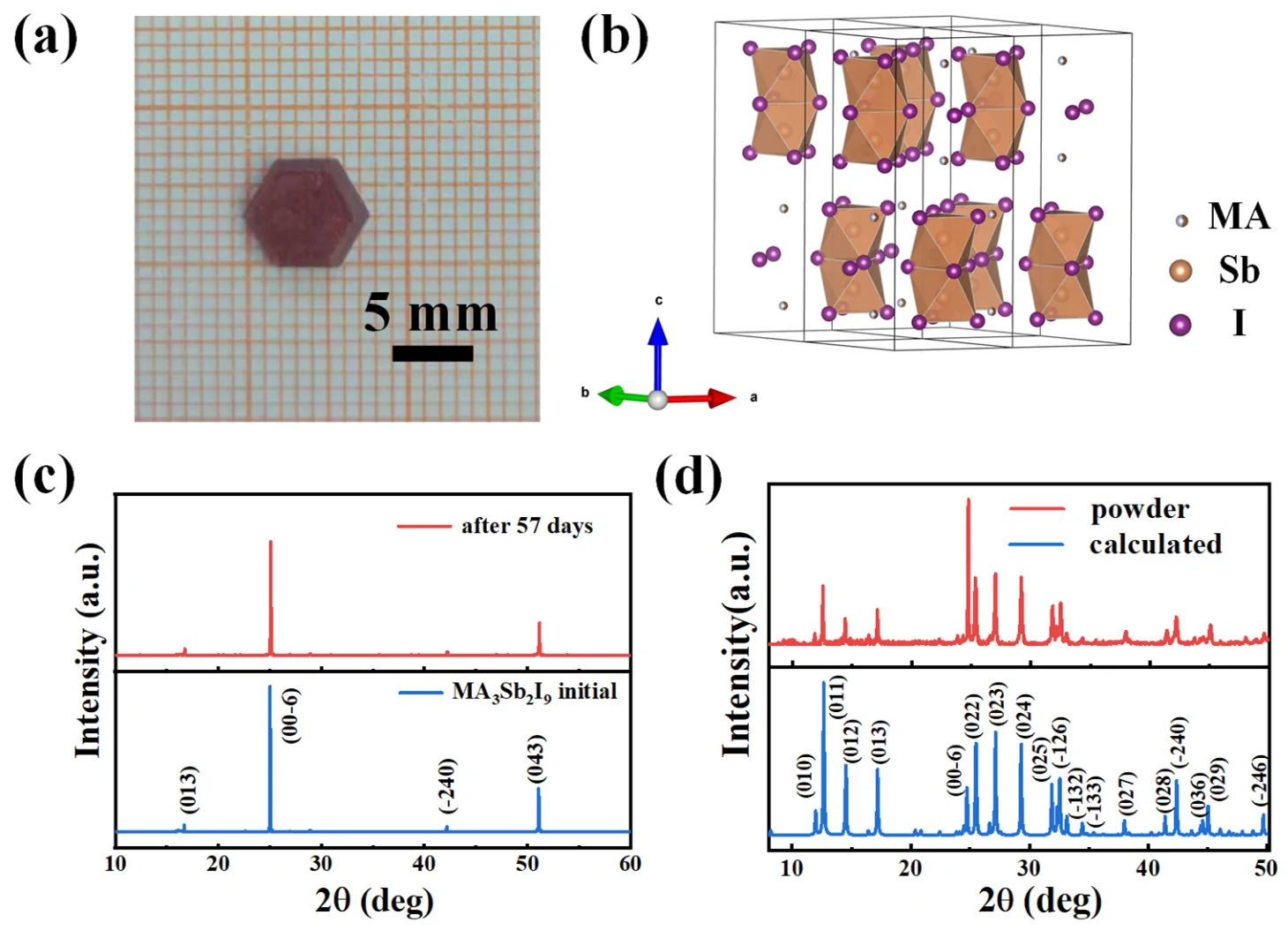
(**a**) Photograph of a ≈ 8 × 7 × 3 mm^3^ dark red MA_3_Sb_2_I_9_ single crystal. (**b**) The unit structures of hexagonal MA_3_Sb_2_I_9_. (**c**) Top-view XRD patterns of as-grown MA_3_Sb_2_I_9_ single crystal and sample stored at 23 °C with 75% RH for 57 days. (**d**) Powder and calculated XRD patterns of MA_3_Sb_2_I_9_ crystal.

A top-view photograph of the well-shaped MA_3_Sb_2_I_9_ single crystal prepared by the seed-induced two-step crystallization method is shown in Figure 2a. The crystal exhibits a dark red color with dimensions of approximately 8 × 7 × 3 mm, and its smooth, crack-free surface indicates high crystalline quality. Figure 2b illustrates the hexagonal unit cell structure of MA_3_Sb_2_I_9_, where Sb^3+^ ions coordinate with I^−^ to form [SbI_6_]^3−^ octahedra that share faces to produce [Sb_2_I_9_]^3−^ dimers. These dimers are uniformly separated by MA^+^ cations rather than directly connected, yielding an isolated 0D arrangement that is a typical feature of 0D perovskites, consistent with previous reports [17]. As shown in Figure 2c, the top-view XRD patterns of the fresh and aged (57 days, 23 °C, ~75% RH) MA_3_Sb_2_I_9_ single crystals are nearly identical. Quantitative analysis of the dominant (00-6) diffraction peak (2θ ≈ 25°) shows a negligible FWHM change, from 0.0384° to 0.0496°, after 57 days of exposure to ambient humidity (~75% RH), confirming that the crystalline integrity is well preserved. The detailed FWHM and intensity variations of four representative peaks are summarized in Table S1. The absence of additional peaks or significant position changes after 57 days confirms good phase stability of the MA_3_Sb_2_I_9_ single crystals under humid conditions. Figure 2d compares the powder and calculated XRD patterns of the MA_3_Sb_2_I_9_ single crystal. The high consistency in peak positions and relative intensities, together with the absence of any additional peaks, confirms that the crystal is free of impurities.

**Figure 3:**
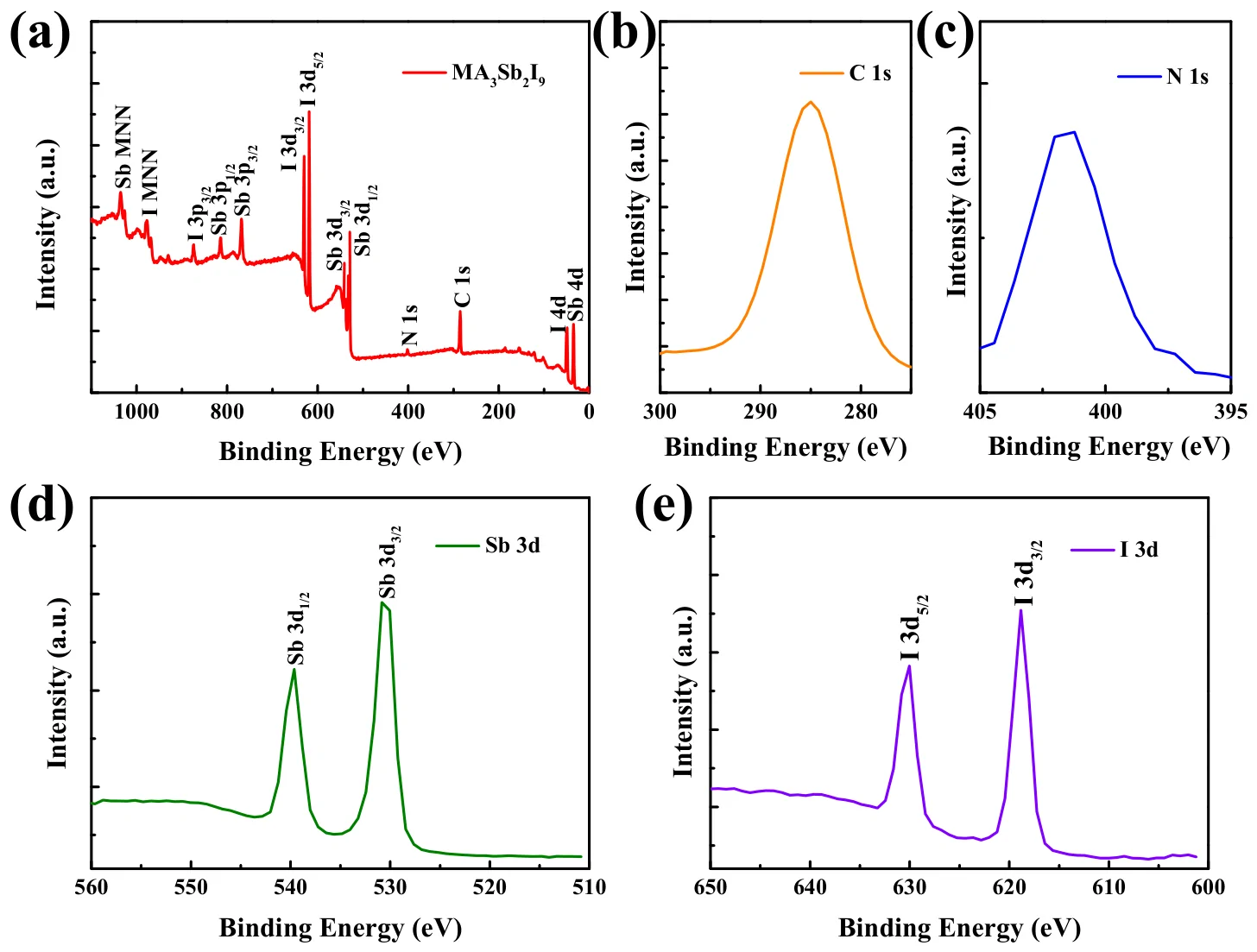
(**a**) XPS spectra of MA_3_Sb_2_I_9_ SC: (CH_3_NH_3_)_3_Sb_2_I_9_. (**b**) C 1s. (**c**) N 1s. (**d**) Sb 3d. (**e**) I 3d.

Figure 3 presents the XPS spectra of the MA_3_Sb_2_I_9_ single crystal. The full-range survey spectrum in Figure 3a unambiguously identifies characteristic peaks of Sb, I, C and N, which are in good accordance with the stoichiometric elemental composition of MA_3_Sb_2_I_9_. No miscellaneous impurity peaks are observed over the entire binding energy range, demonstrating high phase purity of the resultant single crystal. The C 1s peak in Figure 3b centered at ~285 eV and is attributed to the carbon atom of the C-N bond in the MA^+^ cation. The sole N 1s peak located at ~401 eV (Figure 3c) and corresponds to the protonated -NH_3_^+^ group. These two results confirm the intact incorporation of organic MA^+^ into the crystal lattice without decomposition. For the inorganic constituent, the spin–orbit split doublets of Sb 3d (Sb 3d_5/2_ at ~530 eV, Sb 3d_3/2_ at ~540 eV, Figure 3d) correspond to trivalent Sb^3+^ in the halide framework. Meanwhile, the dual peaks of I 3d (I 3d_5/2_ ~619 eV, I 3d_3/2_ ~630 eV, Figure 3e) are characteristic signals of coordinated I^−^ anions. The well-defined sharp peaks of Sb 3d and I 3d verify the formation of an ordered Sb_2_I_9_^3−^ inorganic skeleton. Quantitative analysis based on relative peak areas corrected by sensitivity factors gives atomic ratios of I:Sb = 4.0, I:N = 2.7, and N:Sb = 1.5, which are in good agreement with the nominal values of 4.5, 3.0, and 1.5 for MA_3_Sb_2_I_9_, respectively, confirming the compositional integrity of the crystal. Collectively, XPS characterization verifies the successful fabrication of a high-purity MA_3_Sb_2_I_9_ single crystal with a complete organic–inorganic compositional structure.

**Figure 4:**
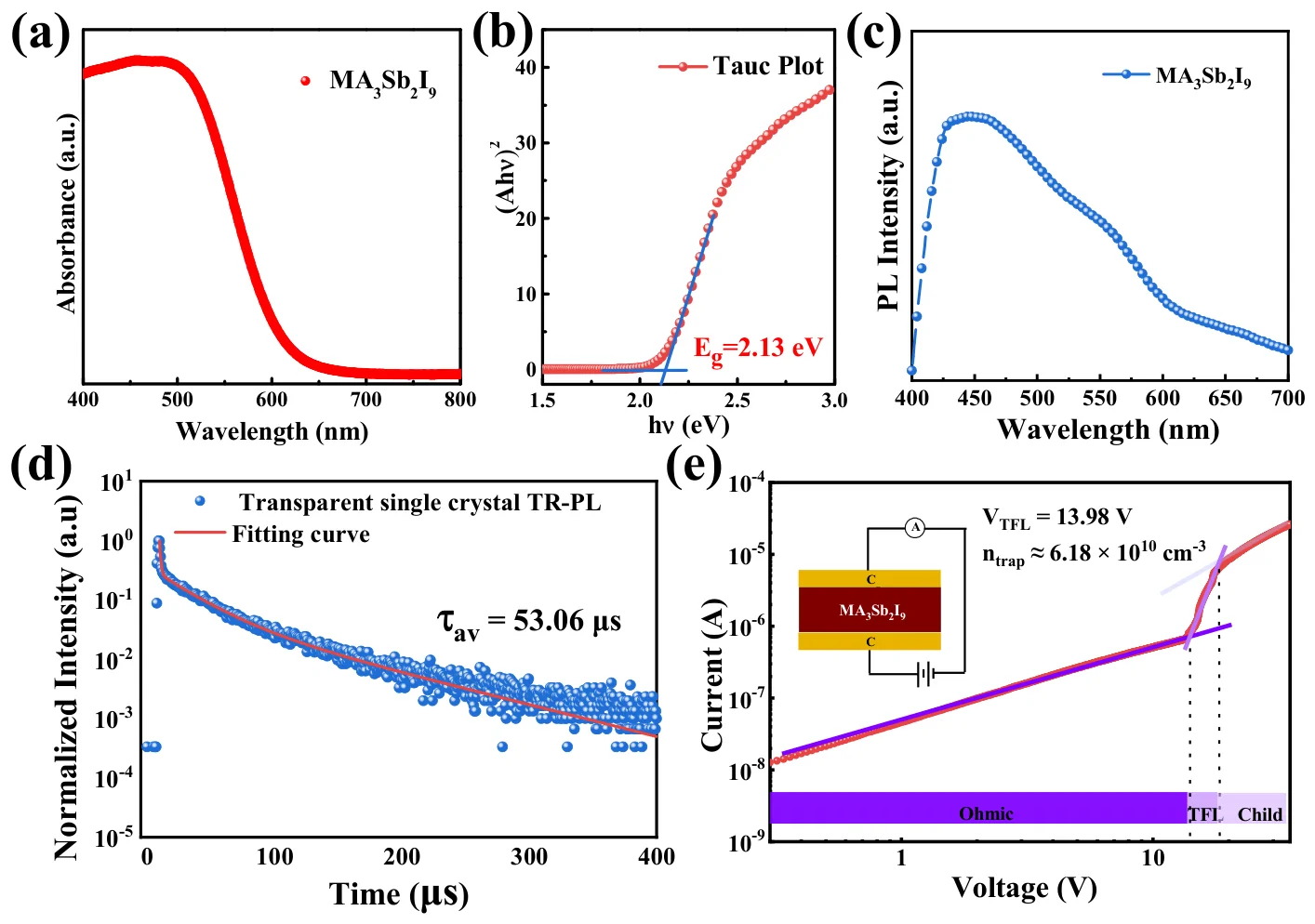
(**a**) UV-vis absorption spectra of the MA_3_Sb_2_I_9_ powder. (**b**) The Tauc plot for MA_3_Sb_2_I_9_. (**c**) PL spectrum of the MA_3_Sb_2_I_9_ single crystal excited at 325 nm (FWHM = 69.4 nm). (**d**) TR-PL spectrum of the MA_3_Sb_2_I_9_ single crystal at 508.9 nm. (**e**) Dark *I-V* plot of MA_3_Sb_2_I_9_ hole-only device using SCLC method. The upper left inset is the device structure.

The UV-vis absorption spectrum (Figure 4a) shows obvious visible-light absorption with the absorption cutoff around 582 nm. The Tauc plot in Figure 4b yields an optical bandgap of E_g_ = 2.13 eV. The steady-state photoluminescence (PL) spectrum (Figure 4c) exhibits a single symmetric emission peak centered at ~508.9 nm with an FWHM of 69.4 nm. Time-resolved PL (TR-PL, Figure 4d) is well fitted by biexponential decay, delivering an average carrier lifetime τ_av_ = 53.06 μs, which is comparable to those reported for high-quality lead-free perovskite single crystals [17,21,22]. It indicates the low density of non-radiative recombination centers inside the crystal, meaning that more photo-generated carriers can survive for potential collection. Figure 4e displays the dark *I-V* characteristic of the hole-only device. By means of a space-charge limited current (SCLC) measurement, the trap-filled limit voltage V_TFL_ = 13.98 V is obtained and the corresponding trap density is calculated as n_trap_ ≈ 6.18×10^10^ cm^−3^. The detailed calculation is provided in the Supplementary Information. The low trap density further confirms the outstanding crystalline quality of the MA_3_Sb_2_I_9_ single crystal.

**Figure 5:**
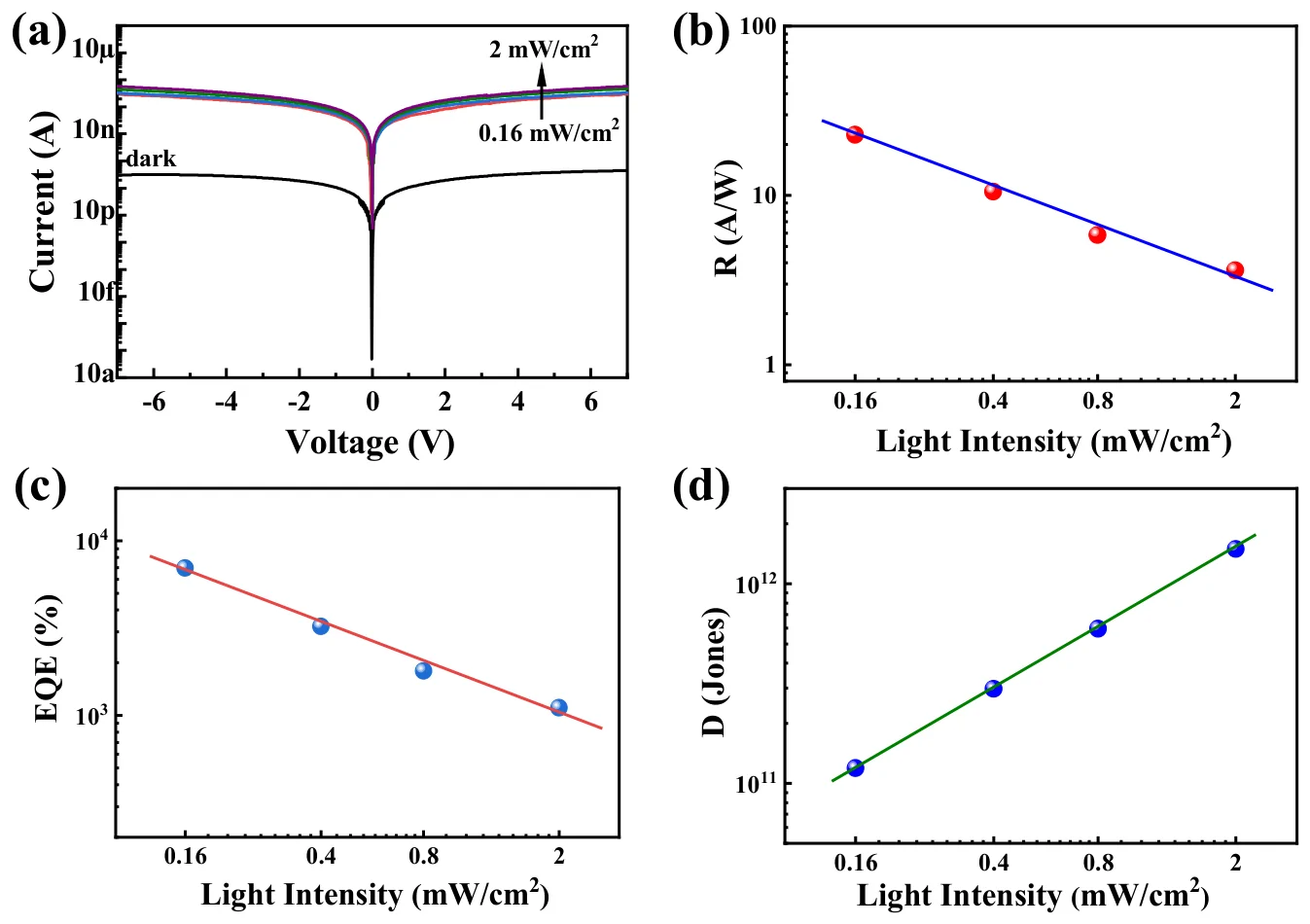
(**a**) *I*-*V* curves of the MA_3_Sb_2_I_9_ single-crystal photodetector in the dark and different light intensities. (**b**) *R*, (**c**) *EQE*, (**d**) *D* for the MA_3_Sb_2_I_9_ single-crystal photodetector at incident light power densities ranging from 0.16 to 2 mW·cm^−2^ at a wavelength of 405 nm under a fixed 7 V bias.

Figure 5 shows the performance of the MA_3_Sb_2_I_9_ single-crystal photodetector measured under 405 nm illumination at a fixed bias of 7 V. *I-V* curves measured in the dark and under different light intensities ranging from 0.16 to 2 mW·cm^2^ are shown in Figure 5a. The device exhibits an ultralow dark current of *I*_dark_ = 4.49 × 10^−10^ A@7 V, and photocurrent increases steadily with the increment of incident light intensity, confirming a typical photoconductive response. The responsivity (*R*) decreases linearly with the increase in incident light intensity, and *R*_max_ = 22.77 A·W^−1^ is obtained at 0.16 mW·cm^−2^. *EQE* shares the same variation trend, with *EQE*_max_ = 6971.92% achieved at the lowest light intensity of 0.16 mW·cm^−2^ (Figure 5c). The equations and detailed parameters for these figures of merit are provided in the Supplementary Information. The observed linear decrease in *R* and *EQE* with increasing light intensity is attributed to the progressive filling of trap states and enhanced bimolecular recombination at higher carrier densities: at low intensity, most photogenerated carriers are collected before recombination, yielding high photoconductive gain, whereas at higher intensity trap-filled limited conduction and increased carrier-carrier scattering reduce the *EQE*. By contrast, specific detectivity (*D*) increases continuously and reaches *D*_max_ = 1.49 × 10^12^ Jones at 2 mW·cm^−2^ (Figure 5d). These results reveal the promising application prospect of the prepared MA_3_Sb_2_I_9_ single crystal for visible-light photodetection. In comparison with previous reports, the device demonstrates competitive performance, as summarized in Table 1. Notably, Equation (S5) was utilized to obtain all reported *D* values in this work, yet the inherent limitations of its underlying assumptions should be taken into account (see Supplementary Information for further details).

**Figure 6:**
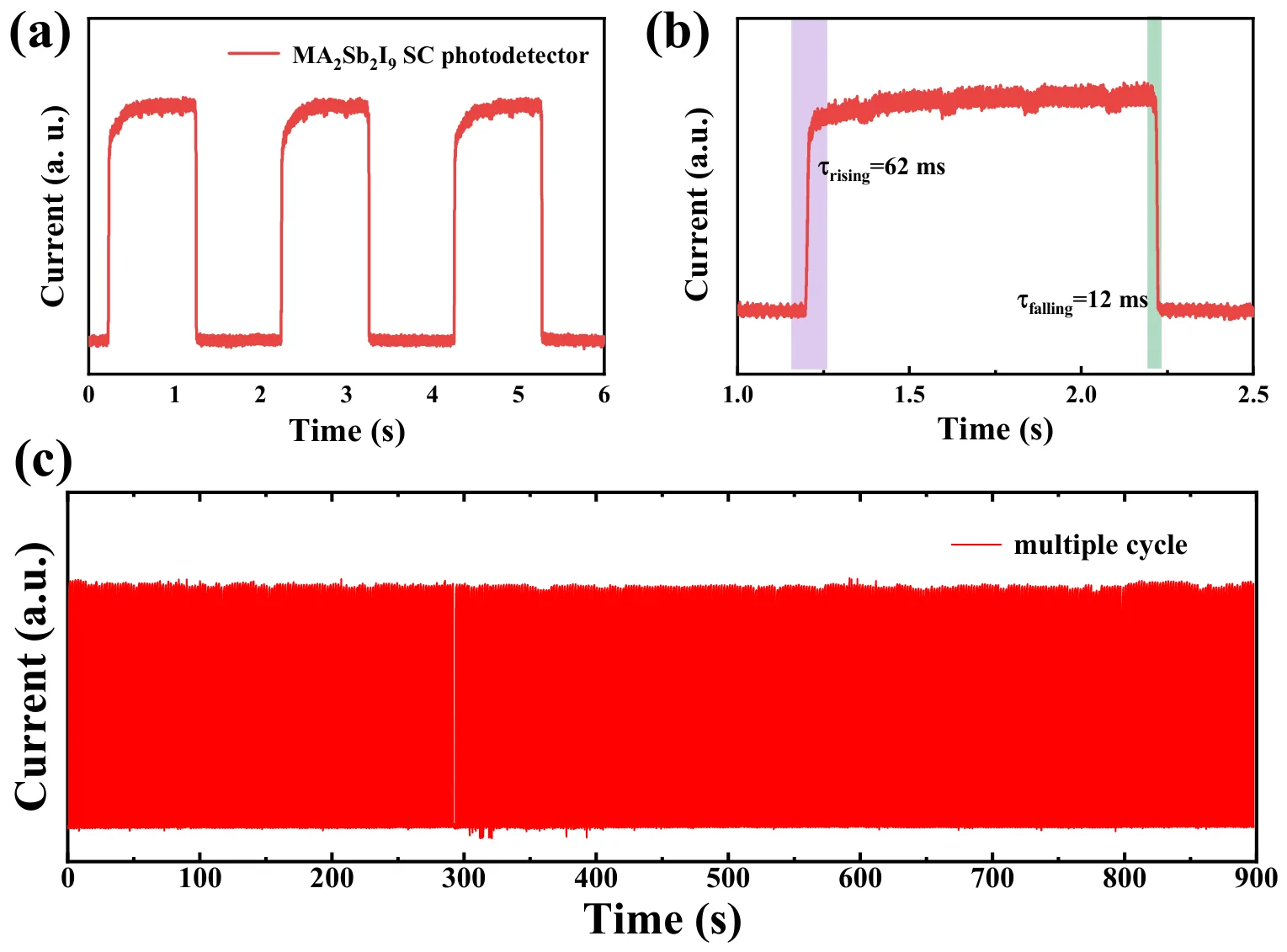
(**a**) Transient photocurrent response over 3 on/off cycles under 405 nm incident light of 8 mW/cm^2^ and 5 V bias. (**b**) Rise and decay times of a single cycle. (**c**) Transient photocurrent response of repeated switching (~450 cycles).

I-*t* curves for transient photocurrent switching of the MA_3_Sb_2_I_9_ single-crystal photodetector collected under 405 nm laser illumination and a 5 V bias are displayed in Figure 6. Three periodic on/off switching cycles are recorded in Figure 6a, where the photocurrent rapidly rises upon light irradiation and decays back to the dark-current level once the light is turned off, revealing well-defined reversible photoconductive switching behavior. From the magnified single-cycle waveform in Figure 6b, the rise time τ_rising_ = 62 ms and fall time τ_falling_ = 12 ms are determined, which confirm fast carrier transportation and superior response speed of the device. As shown in Figure 6c, long-term cyclic tests for ~450 switching cycles reveal no obvious photocurrent degradation, verifying the prominent operational durability and cycling stability of the as-fabricated device.

## 4. Discussion

0D lead-free perovskite single crystals, represented by MA_3_Sb_2_I_9_, have recently attracted attention for X-ray imaging applications owing to their ultralow trap density and high ambient stability. However, research on similar compounds based on Sb is relatively scarce, especially in the field of visible-light photodetection. This is because the isolated [Sb_2_I_9_]^3−^ dimer structure of MA_3_Sb_2_I_9_ may localize the carriers and hinder long-range transport. The present work demonstrates that high-quality MA_3_Sb_2_I_9_ single crystals can be prepared via a seed-induced two-step crystallization method, and the fabricated photodetector achieves promising detection performance.

The measured TR-PL lifetime (53.06 µs) and fast rise time (62 ms) appear inconsistent with the generally expected carrier localization in a 0D framework. This observation can be interpreted as follows: the absence of grain boundaries in the single crystal, combined with the ultralow trap density (~6.18 × 10^10^ cm^−3^), reduces scattering centers to such an extent that carriers can still hop between adjacent [Sb_2_I_9_]^3−^ dimers under an applied bias. This interpretation is consistent with previous reports on 0D MA_3_Bi_2_I_9_, in which low trap densities were also found to mitigate dimensional-originated transport limitations. Nevertheless, direct evidence for the microscopic hopping pathway is not provided in the present study, and the detailed transport mechanism remains to be further investigated.

The present device adopts a lateral coplanar Au/SC/Au geometry, which confines carrier transport near the crystal surface rather than through the bulk. A vertical structure would force carriers to traverse the entire crystal thickness and cross a larger number of MA^+^ spacer layers, further increasing the transport barrier. The light-intensity dependence of *R* and *D* indicates the presence of trap states in the present device. The SCLC measurement gives a bulk trap density as low as ~6.18 × 10^10^ cm^−3^, which is already very small for a single crystal. A previous study has reported that the defect formation energies at the surface and grain boundaries of halide perovskites are lower than those in the bulk [32], resulting in a higher density of surface traps. It is therefore inferred that the trap states responsible for the observed light-intensity dependence are predominantly surface traps and Au/SC interfacial traps, rather than bulk traps. It should be noted that this study is limited by the lack of wavelength-resolved photoresponse data and by the use of a 405 nm LED lamp.

Future work will focus on surface passivation to further reduce trap density, systematic stability evaluation under different bias and temperature conditions, and exploration of 0D MA_3_Sb_2_I_9_ for X-ray detection and optoelectronic synapse applications.

## 5. Conclusions

In summary, high-quality MA_3_Sb_2_I_9_ single crystals were successfully prepared via a seed-induced two-step crystallization method. Systematic characterizations confirm the well-defined 0D crystal structure, high phase purity and excellent ambient stability of the MA_3_Sb_2_I_9_ single crystals. The fabricated MA_3_Sb_2_I_9_ single-crystal photodetector exhibits fast optoelectronic switching behavior with 62/12 ms rise/fall times, as well as stable photocurrent performance over ~450 switching cycles, revealing excellent response kinetics and cycling durability. With balanced high-speed response and robust environmental stability, the MA_3_Sb_2_I_9_ device circumvents the stability limitations of conventional counterparts. This work proves the great application prospect of 0D MA_3_Sb_2_I_9_ perovskites and offers an effective route to develop high-performance lead-free optoelectronic devices.

## Figures and Tables

**Table 1:** Comparison of performance parameters of various materials and device structures for photodetection.

Materials	Device Structure		Wavelength (nm)	Bias (V)	Dark Current (A)	Responsivity (A/W)	Light Intensity (mA/cm^2^)	EQE (%)	D* (Jones)	Response Speed (On/off)	Reference
MA_3_Sb_2_I_9_	Au/SC/Au	H	405	7	4.49 × 10^−10^	22.77	0.16	6971.92	1.49 × 10^12^	62/12 ms	This work
Cs_3_Sb_2_I_9_	Ag/SC/Ag	H	530	1	3 × 10^−9^	~12.2 × 10^−3^	2.34	-	-	46/10 ms	[19]
MA_3_Sb_2_I_9_	Au/microplates/ITO	H	580	3	~10^−12^	15.8 × 10^−3^	1	-	8.1 × 10^11^	2/29 μs	[21]
Cs_3_Bi_2_I_9_	ITO/nanoplates/ITO	H	450	10	<5 × 10^−11^	33.1 × 10^−3^	1.21	9.2	~10^10^	10.2/37.2 ms	[22]
MA_3_Sb_2_I_9_	Au/micro SC/Au	V	1 sun illumination	5	<1 × 10^−9^	~2 × 10^−3^	100	-	-	-	[23]
	ITO/micro SC/ITO	H	1 sun illumination	3	17 × 10^−9^	~2 × 10^−3^	100	-	-	-	
Cs_3_Sb_2_I_9_	Ni/thin film(CVD)/Ni	H	450	2	3.5 × 10^−11^	54.5 × 10^−3^	3.2	-	4.3 × 10^10^	50/30 ms	[24]
Cs_3_Sb_2_Br_9_					9.7 × 10^−13^	~28 × 10^−3^			1.6 × 10^10^	108/56.2 ms	
Cs_3_Bi_2_I_9_	Au/Spiro-MeOTAD/thin film(CVD)/SnO_X_/ITO	V	365	Self-powered	-	1.57	-	-	3.38 × 10^13^	-	[25]
MA_3_Bi_2_I_9_	Au/Spiro-MeOTAD/thin film(CVD)/SnO_2_/ITO	V	UV	Self-powered	-	0.28	-	-	8.8 × 10^12^	-	[26]
Cs_3_Bi_2_I_9_	Au/SC/Si(111)	H	450	-	-	-	-	-	-	1.5 µs	[27]
MA_3_Sb_2_Br_9_	Ag/C60/SC/C60/Ag	H	430	20	8.09 × 10^−9^	0.113		32.7	4.32 × 10^11^	47.1/1162 ms	[28]
Cs_3_Sb_2_Br_9_	Au/SC/Au	H	480	6	2.4 × 10^−12^	2.29	2.55	1.8 × 10^3^	3.77 × 10^12^	<0.2 ms	[29]
Cs_2_AgBiBr_6_	Au/SC/Au	H	550	10	~10^−10^	4.88	7 × 10^−5^	1100	1.22 × 10^13^	8/8.8 µs	[30]
ZnO QDs MoS_2_ NPs	Au/ZnO-MoS_2_/HNP/Au	H	385	10	3.52 × 10^−7^	1.43	34	461	4.50 × 10^10^	3.37/0.35 s	[31]

(a) SC is the abbreviation for single crystal. (b) H represents the horizontal device structure. V represents the vertical device structure.

## Data Availability

The original contributions presented in the study are included in the article/Supplementary Materials. Further inquiries can be directed to the corresponding authors.

## Supplementary Materials

The following supporting information can be downloaded at: https://www.mdpi.com/article/10.3390/nano16211402/s1, Table S1: Variations of representative diffraction peaks of MA_3_Sb_2_I_9_ single crystals before and after 57 days of ambient exposure; Equation S1: Calculation of trap density based on space-charge limited current; Equation S2: *R* calculation; Equation S3: *EQE* calculation; Equation S4: *D** calculation; Equation S5: *D** calculation—simplified. References [33,34,35,36,37,38] are cited in the Supplementary Materials.

## Abbreviations

The following abbreviations are used in this manuscript:

EQE	External quantum efficiency
0D	Zero-dimensional
GBL	γ-Butyrolactone
PL	Photoluminescence
TR-PL	Time-resolved photoluminescence
SCLC	Space-charge limited current
FWHM	Full width at half maximum
R	Responsivity
D	Detectivity
SC	Single crystal
