# Supplementary material for: Zero-Dimensional Lead-Free Perovskite Single Crystals for High-Performance Photodetection
Source: Nanomaterials (Basel). 2026 Nov 1;16(21):1402. doi: 10.3390/nano16211402 (PMC13764741; doi:10.3390/nano16211402)
Supplement: Supplementary file 1 [file nanomaterials-16-01402-s001.zip › nanomaterials-4650290-supplementary.pdf]

Supplementary Information for

Zero-Dimensional Lead-Free Perovskite Single Crystals for High-Performance Photodetection

Jiayu Di[1,*], Shuixiu Lyu[2], Liuyi Li[2], Dawei Wang[1] and Bin Lu[1,*]

[1]School of Computer Science and Artificial Intelligence, Shanxi Normal University, Taiyuan 030031, China

[2]School of Physics and Electronic Engineering, Shanxi Normal University, Taiyuan 030031, China

[*]Correspondence: dijiayu@sxnu.edu.cn (J.D.); lubinsxnu@sina.cn (B.L.)

Keywords: zero-dimensional; lead-free; single crystal; photodetector

Characterization:

XRD was performed with a Bruker D8 Discover X-ray diffractometer with a conventional Cu target X-ray tube set to 40 kV and 40 mA. X-ray photoelectron spectroscopy (XPS) was performed on a photoelectron spectrometer (Thermo ESCALAB 250XI) to investigate the elemental composition and chemical states of the $MA_3Sb_2I_9$ SCs. Absorption spectra were measured using a Perkin-Elmer Lambda 950 UV-Vis-NIR spectrophotometer. Steady-state and TR-PL measurements were taken using a FLS1000 Photoluminescence Spectrometer, with 325 nm excitation wavelength. EDS spectroscopy mapping were recorded by Oxford (X-ACT) scanning electron microscope. All material characterizations were measured in the air without encapsulation.

Device Fabrication and Characterization

Hole-only devices were made by coating carbon on two opposite surfaces of the single crystals as electrodes. SCLC measurements were performed using a Keithley 4200 instrument. The $MA_3Sb_2I_9$ single crystals were used to make planar photodetectors. Au/Perovskite single-crystal/Au horizontal structure devices (Au is 100 nm, effective area of the device is 0.008 mm^2) are used to measure the 405 nm light response. The *I*-*V* characteristics of the Photodetector were measured using a Keithley 4200 source meter excited by 405 nm LED lamp (Xi'an Zhuoli Hanguang Co., Ltd.). All measurements are performed at room temperature.

Table S1. Variations of representative diffraction peaks of $MA_3Sb_2I_9$ single crystals before and after 57 days of ambient exposure.

**(013)**	**2θ (deg)**	**Intensity (counts)**	**FWHM (deg)**
Initial	16.6966	2102	0.0368
After 57 days	16.7578	1923	0.0491
**(00-6)**	**2θ (deg)**	**Intensity (counts)**	**FWHM (deg)**
Initial	25.0248	45159	0.0384
After 57 days	25.0860	35286	0.0496
**(-240)**	**2θ (deg)**	**Intensity (counts)**	**FWHM (deg)**
Initial	42.1711	1586	0.0512
After 57 days	42.2323	1127	0.0578
**(043)**	**2θ (deg)**	**Intensity (counts)**	**FWHM (deg)**
Initial	51.0912	13436	0.0582
After 57 days	51.1525	10041	0.0638

SCLC measurement

The trap density of the $MA_3Sb_2I_9$ single crystal was evaluated by SCLC measurement using the hole-only Au/SC/Au device. As the applied voltage increases, the current-voltage (*I-V*) curve undergoes three distinct regions: an ohmic region at low voltage, followed by a trap-filled limit (TFL) region, and finally a trap-free space-charge-limited region at high voltage. The transition from the ohmic region to the TFL region corresponds to the trap-filled limit voltage (V_{TFL}), which is identified as the inflection point in the *I-V* curve. The trap density (n_{trap}) is calculated according to **Equation S1:**

$$n_{\mathrm{trap}} = \frac{2\varepsilon\varepsilon_0 V_{TFL}}{qL^2} \quad (1)$$

where $\varepsilon_0 = 8.854 \times 10^{-12}$ F/m is the vacuum permittivity, $\varepsilon \approx 40$ is the relative dielectric constant of $MA_3Sb_2I_9$ at room temperature[33], $q = 1.602 \times 10^{-19}$ C is the elementary charge, and $L \approx 1$ mm is the thickness of the $MA_3Sb_2I_9$ single crystal used for the hole-only device. With $V_{TFL} = 13.98$ V, the trap density is calculated to be $n_{\mathrm{trap}} \approx 6.18 \times 10^{10}$ cm^{-3}.

Photodetector performance metrics

The responsivity, external quantum efficiency, and detectivity of the $MA_3Sb_2I_9$ single-crystal photodetector were calculated using the following equations:

1) Responsivity (*R*):

the ratio of the photogenerated current of device to the input optical power, expressed in A/W or V/W.

Equation S2

$$R = \frac{I_{\mathrm{ph}}}{P_{\mathrm{in}} \times A} = \frac{I_{\mathrm{illuminated}} - I_{\mathrm{dark}}}{P_{\mathrm{in}} \times A} \tag{2}$$

where I_{ph} is photogenerated current, $I_{\mathrm{illuminated}}$ is output photocurrent, I_{dark} is dark currrent, P_{in} is incident light powder, A is device efficient area.

2) External quantum efficiency (*EQE*):

the ratio of the number of generated electrons collected by the electrodes at both ends to the number of incident photons, which can be calculated by the *R* and incident photon energy.

Equation S3

$$EQE = R \cdot \frac{hc}{e\lambda} \tag{3}$$

where h is Planck's constant, c is the vacuum speed of light, e is the unit charge, λ is incident light wavelength. In the photoconductive Metal-Semiconductor-Metal (MSM) mode, an *EQE* exceeding 100% is expected because photoconductive gain allows a single absorbed photon to generate multiple charge carrier cycles in the external circuit. This value thus reflects the combined effect of internal quantum efficiency and photoconductive gain, rather than the internal quantum efficiency alone.

3) Detectivity (*D*) :

D is used to measure the detection ability of the device. The unit is Jones or cm·Hz$^{1/2}$·W^{-1}.

Equation S4

$$D=\frac{\sqrt{A\cdot\Delta f}}{NEP} \quad (4)$$

Notably, a direct noise spectral measurement was not conducted in the present study. When the noise in the device is mainly dark current, it can be further simplified as:

Equation S5

$$D=\frac{R}{\sqrt{2eJ_{\text{dark}}}} \quad (5)$$

where Δf is measurement bandwidth, *NEP* is noise equivalent power, J_{dark} is dark current density.

Equation S5 is a simplified inference derived from the full definition provided in Equation S4. It establishes the proportional relationship between specific *D* and *R* when device noise is dominated by dark-current-related contributions. With increasing illumination intensity, enhanced carrier recombination causes *R* to decrease at higher irradiance; nevertheless, the signal-to-noise ratio can still be further improved, yielding higher specific *D*. Although Equation S5 is widely employed for practical calculations in numerous photodetector-related publications[34-38], one should keep in mind the inherent limitations of its underlying assumptions.
